# Liquid Biopsy in Pituitary Neuroendocrine Tumors—Potential Biomarkers for Diagnosis, Prognosis, and Therapy

**DOI:** 10.3390/ijms26094058

**Published:** 2025-04-25

**Authors:** Ligia Gabriela Tataranu

**Affiliations:** 1 Neurosurgical Department, Carol Davila University of Medicine and Pharmacy, 020022 Bucharest, Romania; ligia.tataranu@umfcd.ro; 2 Neurosurgical Department, Bagdasar-Arseni Clinical Emergency Hospital, 041915 Bucharest, Romania

**Keywords:** liquid biopsy, PitNET, pituitary tumors, circulating tumor DNA, cell-free RNA, circulating tumor cells, exosomes

## Abstract

Pituitary neuroendocrine tumors (PitNETs) are slow-growing neoplasms with various clinical presentations, often leading to diagnostic challenges. While neuroimaging assessment and histopathological evaluation remain the gold standard for diagnosis, emerging research highlights the potential of liquid biopsy, mainly through the analysis of circulating non-coding RNAs (ncRNAs), as a promising diagnostic and prognostic tool. Recent studies have demonstrated distinct expression profiles in different types and subtypes of tumors, with implications in assessing tumor aggressiveness and predicting treatment response. The current article summarizes data about potential biofluid markers implicated in PitNET development, mainly circulating tumor DNA (ctDNA), cell-free RNAs (cfRNA), circulating tumor cells (CTCs), and exosomes. Many studies on genetic and molecular markers in PitNET tissue samples provide exciting information about tumor biology, but to date, specific studies on liquid biopsy biomarkers are still few. Over the past years, a certain understanding of the mechanisms involved in pituitary tumorigenesis has been gained, including the landscape of genomic alterations, changes in epigenetic profile, crucial molecules involved in specific signaling pathways, and non-coding RNA molecules with critical roles in malignant transformation. Genetic and molecular characterization of the PitNETs could help distinguish between functional and non-functional PitNETs, different types and subtypes of pituitary tumors, and invasive and non-invasive forms. Further studies are required to identify and validate innovative biomarkers or therapeutic targets for treating PitNET. Integrating liquid biopsy into clinical workflows could revolutionize the management of pituitary adenomas, enabling more personalized and less invasive diagnostic and therapeutic strategies.

## 1. Introduction

Pituitary neuroendocrine tumors (PitNETs), formerly known as pituitary adenomas, are a complex and heterogeneous group of well-differentiated tumors arising from the anterior lobe of the pituitary gland. They account for about 10–15% of all intracranial tumors and are the second most common primary intracranial neoplasms in adults [1]. These tumors can exhibit various clinical behaviors. They are mostly benign, but 30–40% of surgically treated cases are invasive into the surrounding structures like the sphenoid sinus or the cavernous sinus, and this is the main limitation for maximal safe resection [2,3,4]. These invasive tumors cannot be removed entirely and are associated with a higher recurrence rate. Invasiveness can be evaluated by preoperative neuroimaging assessment, intraoperative evaluation, and postoperative pathological examination. About 10% of PitNETs with invasive behavior are clinically aggressive [1]. According to the European Society of Endocrinology Clinical Practice Guidelines, the diagnosis of an aggressive PitNET should be considered in patients with a radiologically invasive tumor and unusually rapid tumor growth rate or clinically relevant tumor growth despite optimal standard therapies (surgery, radiotherapy, and conventional medical treatments) [5]. Only 0.1–0.2% of PitNET can be associated with craniospinal and/or systemic metastases, but these metastatic lesions generally do not become poorly differentiated. Therefore, the term “metastatic PitNET” is preferred instead of the former “pituitary carcinoma” terminology.

The molecular factors driving PitNETs to become invasive are not yet fully understood. Yang and Li studied the molecular network basis of invasiveness and proposed the following stages in the invasive transformation of PitNETs: (1) the overexpression of Hypoxia-inducible Factor-1 alpha (HIF-1α), which facilitates the adaptation of tumor cells to low oxygen and nutrient availability and promotes tumor growth, invasion, and migration; (2) the overexpression of Vascular Endothelial Growth Factor A (VEGFA), which has proved to be a key factor in angiogenesis but seems to have a more direct role in tumorigenesis and invasiveness; (3) epithelial-to-mesenchymal transition (EMT) triggered by Pituitary Tumor-transforming Gene (*PTTG1*), known as an oncogene with an upregulation effect on VEGF; (4) the overexpression of Matrix Metalloproteinases (MMPs), which lead to the degradation of the basement membrane and extracellular matrix (ECM) and promote invasiveness [3,6].

Based on their ability to produce hormones, PitNETs are classically divided into clinically functioning and non-functioning tumors. Functioning PitNET causes specific conditions related to hormonal hypersecretion. Thus, lactotroph tumors (30–50% of all PitNET) secrete high levels of prolactin (PRL), leading to amenorrhea–galactorrhea syndrome in females and impotence in males, with infertility. Somatotroph tumors (15–20% of all PitNET) cause acromegaly in adults and gigantism in children through growth hormone (GH) hypersecretion. Most corticotroph tumors (15% of functioning PitNET) have an excessive adrenocorticotropic hormone (ACTH) secretion, resulting in Cushing’s disease. Thyrotroph tumors (0.5–1.5% of all PitNET) are responsible for central hyperthyroidism due to elevated levels of thyroid hormones. Gonadotroph tumors represent 15–40% of all PitNET, but they are very rare and functionally inactive. The hypersecretion of β-follicle-stimulating hormone (FSH) and/or β-luteinizing hormone (LH) does not commonly produce a clinical syndrome [7]. There are also plurihormonal tumors. Non-functioning PitNETs (NF-PitNETs) are usually diagnosed due to symptoms related to the mass effect or compression of the normal pituitary with panhypopituitarism. Approximately 30% of these tumors show no immunostaining for any hormone, while more than 45% stain for gonadotropins or their subunits [8]. Some of these tumors exhibit aggressive behaviors, invading surrounding anatomical structures [9].

The accuracy of the diagnosis of pituitary tumors has improved by routine use of immunohistochemistry (IHC) for pituitary transcription factors (PIT1—Pituitary-specific positive transcription factor 1, TPIT—T-box pituitary transcription factor, SF1—Steroidogenic factor 1, GATA3, and ERα—Estrogen receptor alpha), producing hormones and other biomarkers, which is endorsed in the 5th Edition of the WHO Classification of Endocrine and Neuroendocrine Tumors. This classification distinguishes adenohypophyseal tumors as a complex family of epithelial neuroendocrine neoplasms with specific cell lineages and subtypes based on their morphologic, molecular, and clinical characteristics [10]. It helps the clinician apply individualized patient treatment and follow-up and recognize the potentially aggressive forms of diseases early. The French Five-Tiered Prognostic Classification takes into consideration the tumor diameter, the IHC type, the invasion, and the proliferative markers (KI-67 index, mitotic count, and p53 positivity) and stratifies the tumors into five different grades according to their invasiveness and proliferative potential [4,11]. Statistical analyses have validated the prognostic value of this classification, and the European Pituitary Pathology Group (EPPG) recommended including the French Five-Tiered grading in the integrated diagnosis [12].

These tumors’ diagnosis, classification, and treatment are based on clinical evaluation, neuroimaging, hormonal profiling, and pathological analysis of solid tumor tissue obtained through surgery. While tissue biopsy remains the gold standard for acquiring reliable information on PitNETs, it does have some limitations. It is an invasive procedure with some difficulties and risks. Due to tumor heterogeneity, the sample may not always be representative of the lesion. It is not convenient to repeat the tissue biopsy for adequate monitoring of disease progression and therapy response. Given these limitations, the necessity to identify additional clinically useful biomarkers is obvious.

Liquid biopsy has gained increasing interest as a promising alternative for rapid and minimally invasive diagnostic, prognostic assessment, clinical course monitoring, and treatment response evaluation. Liquid biopsy permits the detection of tumor-derived circulating biomarkers in blood or other bodily fluids, offering insights into the tumor’s molecular profile. Due to the high vascularization of the pituitary gland, there is a strong connection between its cells and the circulatory system. In this area, there is no blood–brain barrier. The results from liquid biopsy can complement traditional neuroimaging and hormonal tests, offering a more comprehensive understanding of the disease. This approach enhances precision medicine by incorporating molecular data into treatment strategies [13]. It presents several key advantages. It offers a minimally invasive approach. It is a laboratory test performed on a fluid sample (blood sample, saliva, urine, etc.), which is safe and easy to collect. It is particularly appropriate for those who may not be able to undergo traditional surgical biopsies. Also, it can be repeated as needed, making it possible to monitor disease progress over time without the risks of multiple invasive procedures. Additionally, it can give a more complete image of tumor genetic heterogeneity that might not be available through traditional biopsy methods. However, the clinical utility of liquid biopsy in PitNET is currently limited. There are specific challenges related to the isolation and processing of biomarkers, which sometimes require advanced technologies; the sensitivity and specificity are not always optimal, and the cost is another limitation. On the other hand, the method may not capture the tumors’ entire molecular and genetic complexity [14].

**Figure 1:**
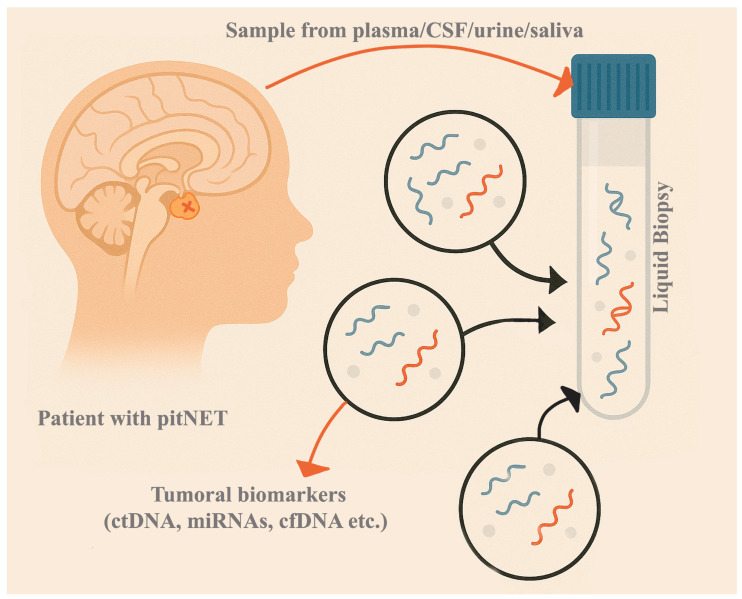
An illustration regarding the liquid biopsy in PitNETs.

The present narrative review aims to summarize the existing knowledge and recent advancements in identifying potential liquid biopsy biomarkers for PitNET. Characterizing the circulating molecular profile can become a viable option for an accurate and early diagnosis, optimally predicting the prognosis, and monitoring each patient’s disease course and treatment response. Eventually, an accurate disease status can be outlined only by the integration of multiple biomarkers, sampling approaches, and datasets [15]. A summary of the current article is presented in Figure 1.

## 2. Liquid Biopsy Potential Biomarkers in PitNETs

The main components of liquid biopsy include circulating tumor cells (CTCs), circulating tumor DNA (ctDNA), cell-free RNA (cfRNA), and exosomes. Although each component will be further discussed in detail, a summary of their characteristics, as well as advantages and disadvantages, is presented in Table 1.

### 2.1. Circulating Tumor Cells (CTCs)

Circulating tumor cells are detached from tumor tissue and disseminated into body fluids, including blood. However, many CTCs change phenotype after EMT, with consequent loss of cell-surface epithelial markers, which are used for detecting these cells. With technological advances and innovations, these cells can be isolated and used for characterizing and monitoring solid tumors by genome, proteome, transcriptome, and secretome analysis [16]. Despite their potential role as a diagnostic marker, CTCs are extremely rare in the bloodstream in cases of PitNETs, which are generally small tumors. CTCs were detected in benign tumors such as PAs (Pituitary Adenomas) in early-stage disease, and not only in late-stage malignant tumors with apparent distant metastases [17]. There is ongoing research in this field, but the clinical utility of CTCs remains unclear [18].

### 2.2. Circulating Tumor DNA (ctDNA)

Cell-free DNA (cfDNA) is fragmented DNA (>500 bp) originating from genomic DNA that is released into the biological fluids from both normal and abnormal cells [19,20,21]. There are also smaller fragments of DNA (<100 bp) released into the blood circulation from tumor cells by necrosis, apoptosis, and active secretion [19]. They are called circulating tumor DNA (ctDNA). Tumor-specific genetic and epigenetic alterations or molecular biomarkers distinguish ctDNA from normal cfDNA fragments. Sometimes, ctDNA is released within extracellular vesicles, which protect the genetic material from being broken down by plasma nucleases. Because of its short half-life (16 min to 2.5 h), ctDNA allows for a dynamic, real-time assessment of tumor evolution through serial sampling [16,22]. Malignant tumors discharge higher amounts of ctDNA into the bloodstream than benign ones. The ctDNA levels decrease after tumor resection and increase with its recurrence, which could permit close disease monitoring and orient clinical decisions [14,18,23,24].

Given that ctDNA contains the same genetic and epigenetic variations as DNA from tumor cells, it is possible to study not only the genetic landscape of primary tumors but also intratumoral clonal heterogeneity [25]. Various tumor-specific genetic alterations or epigenetic marks could be identified, thus serving as a non-invasive tool to assess the genetic profile of PitNETs. The sequencing of a single gene, a gene panel, the whole exome, or even the whole genome could be performed. The modifications revealed by liquid biopsy using ctDNA are quite consistent with conventional genetic testing through tissue biopsy [14]. However, to date, few studies have been conducted to detect and study ctDNA in pituitary tumors [23]. The detection and sequencing of ctDNA in pituitary tumors remains to be validated in clinical practice. Most of the data regarding PitNET are available from tumoral tissue analysis.

#### 2.2.1. Genetic Alterations

Despite technological advancements, the landscape of genomic alterations in PitNETs is yet to be clarified. They are generally benign tumors with high genomic and genetic heterogeneity, and the determination of potential biomarkers is challenging. Particular mutant genotypes confer a selective advantage to specific subclonal cells so that they have greater chances of surviving and reproducing than normal cells and eventually become dominant in a local tissue environment [26].

Familial PitNETs represent less than 5% of all pituitary tumors. They occur in a syndromic or hereditary setting and result from well-defined, germline genetic abnormalities. Germline mutations are generally associated with more invasive, aggressive, and drug-resistant tumors.

The combined occurrence of PitNET, parathyroid adenomas, and neuroendocrine tumors characterizes Multiple Endocrine Neoplasia Type 1 (MEN1) syndrome. It is due to an inactivating mutation of the tumor suppressor gene *MEN1*, which encodes a ubiquitously expressed transcription cofactor of cyclins and participates in G1/S checkpoint regulation [27].

Multiple Endocrine Neoplasia Type 4 (MEN4) syndrome has a MEN1-like phenotype, but loss-of-function mutations in the cyclin-dependent kinase inhibitor 1B (*CDKN1B*) gene are found (encodes p27^Kip1^, a CDK inhibitor that prevents cell cycle progression from G1 to S phase, thus acting as a tumor suppressor gene) [28].

Carney Complex is associated with spotty pigmentation of the skin, multiple cardiac and extracardiac myxomas, and endocrine tumors, including GH-secreting PitNETs. Inactivating mutation of the *PRKAR1A* gene has been shown to cause the syndrome. This gene encodes the R1α regulatory subunit of protein kinase A (PKA), inhibiting the binding of cAMP and activating the kinase activity. The loss of R1α increases PKA responsiveness to cAMP, and the pro-mitotic cAMP pathway is activated, promoting somatotroph tumorigenesis [28].

The “3Pas” syndrome combines pituitary adenoma with pheochromocytoma/paraganglioma (PPGL) and is due to activating mutations of the genes encoding succinate dehydrogenase (SDH) subunits and the SDH complex assembly factor 2 protein (SDHAF2). These mutations might impair the electron transfer chain of the mitochondria, mimicking hypoxia, and lead to the accumulation of succinate. An increase in HIF-1α induces resistance to apoptotic signals and enhances glycolysis, promoting tumorigenesis [27]. Commonly, there are PRL-secreting, GH-secreting, or NF-PitNETs, frequently macroadenomas with an aggressive clinical course and with poor therapeutic response.

DICER1 syndrome results from inactivating germline mutations in the *DICER1* gene, which encodes a cytoplasmic endoribonuclease that cleaves precursor microRNA (miRNA) into functional miRNA that downregulates targeted mRNAs, thereby modulating cellular protein production. This condition predisposes to unusual tumors, both benign and malignant, such as pituitary blastoma, pleuropulmonary blastoma, or embryonal tumors. The symptomatology is commonly evident within the first year of life [26].

X-linked acrogigantism (X-LAG) syndrome consists of microduplications of a 500 bp region on chromosome X, where the gene-encoding G protein-coupled receptor 101 (*GPR101*) is located. The overexpression of the *GPR101* gene leads to the activation of the stimulatory G protein complex. It activates adenylate cyclase, increasing cyclic adenosine monophosphate (cAMP) production and directly affecting somatotroph cell proliferation and GH secretion. Patients display GH-secreting or GH-PRL-secreting tumors or pituitary hyperplasia with an early onset of clinical features and a poor response to treatment [28].

The familial isolated pituitary adenoma (FIPA) syndrome is characterized by early-onset and usually aggressive GH-secreting PitNETs, often resistant to treatment. The common cause of hereditary isolated cases is inactivating tumor suppressor gene mutations encoding the aryl-hydrocarbon receptor-interacting protein (AIP). The AIP is a co-chaperone able to bind to different partners. Decreased AIP expression contributes to pituitary tumorigenesis by interacting with cAMP/PKA pathway components and acting upstream of cAMP production and impaired stabilization of aryl hydrocarbon receptor (AhR). It is a transcription factor known for mediating the effects of environmental toxins and is involved in regulating the cell cycle and differentiation. Moreover, *AIP* mutations alter interactions with zinc finger protein 1 (ZAC1), which acts as a transcription factor and coregulator in the pituitary cells [28,29].

A summary of the aforementioned genetic alterations and their main characteristics is rendered in Table 2.

Sporadic PitNETs account for 95% of cases. Non-heritable somatic mutations are found in variable proportions in pituitary tumors and are involved in the tumorigenesis of certain PitNET types.

In the somatotroph tumors, gain-of-function somatic mutations of the gene encoding the guanine nucleotide-binding protein alpha subunit (GNAS) are known to occur in 15–40% of GH-secreting tumors [29,30]. The mutations in the *GNAS* gene are the only ones to be causally linked to somatotroph tumor pathogenesis [28]. The gene (historically called gsp oncogene) encodes the α subunit of the stimulatory heterotrimeric guanine nucleotide-binding proteins (G proteins) (Gsα). The α subunit binds guanine nucleotides and acts as a GTPase. As a result of mutations, an aberrant GTPase activity appears, as well as increased levels of cAMP production, increased PKA activity, activation of CREB (cAMP-response element binding protein), and transcription of the gene-encoding GH. Ultimately, these processes increase somatotroph cell proliferation, GH synthesis, and release. However, the *GNAS*-mutated tumors are often smaller, less invasive, and respond better to somatostatin analogs, probably because there are intracellular regulatory mechanisms able to counteract the activation of the cAMP pathway [28]. One type of *GNAS* mutation (p.R201C) was also detected in corticotropinomas and non-secreting PitNETs.

Various less common somatic mutations are potentially associated with sporadic somatotroph PitNET. *MEN1* and *AIP* gene mutations can also be found in sporadic forms of disease. Upregulated expression of *PTTG1* (Pituitary tumor-transforming gene 1, securin) induces genetic instability by inhibiting chromatid separation during mitosis and increasing cell proliferation. This proto-oncogene is associated with aggressive tumors.

STAT 3 (Signal Transducer and Activator of Transcription type 3) is overexpressed in somatotroph adenomas, leading to GH hypersecretion and resistance to therapy. It is a transcription factor that transduces signals from cell surface receptors (cytokines, growth factors, etc.) to the nucleus, where it regulates the expression of genes involved in critical cellular processes such as survival, differentiation, immune response, and apoptosis.

*CDH23* (cadherin-related 23) mutations are associated with sporadic PitNET in 12% of cases, mainly with somatotroph adenomas [27]. CDH23 is a calcium-dependent cell–cell adhesion glycoprotein. The mutation of *CDH23* (which can also be found as a germline mutation) causes an amino acid substitution in the calcium-binding motif of the extracellular domain of CDH23 and is an inactivating mutation predicted to impair cell–cell adhesion [31]. CDH23 regulates the Wnt (translated products of the WNT gene) signaling pathway, which plays an important role in the differentiation and proliferation of pituitary cells and pituitary tumorigenesis.

SLC20A1 (Solute Carrier Family 20 Member 1, phosphate transporter 1) overexpression is correlated with tumor size, invasive behavior, and tumor recurrence of somatotroph adenomas. SLC20A1 is a transporter that absorbs phosphate from interstitial fluid for use in cellular functions such as metabolism, signal transduction, and nucleic acid and lipid synthesis. It may be associated with the activation of the Wnt/β-catenin signaling pathway [32].

*PRDM2* (PR Domain Zinc Finger Protein 2) is a tumor suppressor gene associated with somatotroph adenomas. The absence of PRDM2 is likely to be involved in the tumorigenesis of somatotroph adenomas by regulating c-Myc oncogene, which is involved in cellular metabolism and proliferation [33].

Somatostatin receptor (SSTR1-5) and dopamine receptor (DRD1-5) subtype expressions were correlated with treatment response in somatotropinomas. Thus, it has been proposed that a decreased expression of SSTR1, SSTR2, DRD4, and DRD5 may be associated with a poor response to SSAs (somatostatin analogues) treatment [34].

In the corticotroph PitNET, somatic mutations of the gene encoding the *USP8* (ubiquitin-specific peptidase 8) are found in 20–60% of cases [28,29]. USP8 is a deubiquitinating enzyme that removes ubiquitin molecules from substrates, thereby preventing the proteasomal degradation of the substrate. If the substrate is an oncogene or proto-oncogene, it can promote tumorigenesis. Gain-of-function mutations in *USP8* increase the deubiquitination of EGFR (epidermal growth factor receptor), which inhibits its degradation. EGFR is overexpressed in these cases and promotes the initiation and progression of tumors by activating multiple signaling pathways, inhibiting the tumor suppressor p53 and other apoptotic proteins [29,35]. The high EGFR levels stimulate *POMC* (proopiomelanocortin) gene transcription and enhance ACTH synthesis. *USP8*-mutated tumors are more common in females and are characterized by an earlier onset, a smaller size, and increased ACTH production. They have a greater probability of surgical remission and a higher risk of recurrence. Somatostatin receptor type 5 (SST5) has increased expression in these tumors, which probably implies a better response to pasireotide treatment [36].

At much lower rates, somatic *USP48* and *BRAF* mutations have been described in USP8 wild-type corticotroph PitNETs, glucocorticoid receptor *NR3C1*, and *TP53* mutations [29]. Mutations in the protein-coding gene of deubiquitinase *USP48* were reported in 23% of corticotroph PitNET, and the pathogenetic mechanism involved is the NF-κB signaling pathway, implicated in the CRH-induced transcriptional activation of the *POMC* gene, which increases plasma ACTH levels [37]. Mutations in *BRAF* oncogene appear in 16% of corticotropinomas. In these tumors, an enhancement of the promoter activity and transcription of the gene-encoding POMC is involved, with the overproduction of ACTH [37].

*TP53* is a gene encoding the p53 tumor suppressor protein, with functions in cell cycle arrest, aging, DNA repair, and apoptosis. *TP53* mutations in functional corticotroph PitNET were analyzed. Variants altering protein function have been found in 15% of cases of USP8 wild-type tumors, but there were no *TP53* mutations in *USP8* mutant tumors. *TP53* mutant tumors were more extensive and more invasive, with worse disease outcome [38].

Less common genetic variations potentially associated with sporadic corticotroph PitNET was identified, including *HSP90* (Heat Shock Protein 90) gene overexpression, *Brg1* and *HDAC2* (Histone Deacetylase 2) deficiencies, loss-of-function mutations of *CABLES1* (CDK5 and ABL Enzyme Substrate 1) tumor suppressor gene, decreased expression of *SFRP2* (Secreted Frizzled—Related Protein 2) tumor suppressor gene [27]. *DICER1* gene variants may contribute to the pathogenesis of nonsyndromic corticotropinomas.

In the PRL-secreting PitNET, an *SF3B1* mutation has been identified in a subset of tumors. *SF3B1* encodes subunit 1 of the splicing factor 3b complex. This mutation causes aberrant splicing of estrogen-related receptor gamma (ESRRG), which results in stronger binding of pituitary-specific positive transcription factor 1 (Pit-1), leading to abnormal PRL secretion and tumorigenesis in prolactinomas [39].

High Mobility Group A (HMGA) proteins are DNA architectural factors that play key roles in chromatin architecture, gene transcription, and replication. These nuclear proteins exert their oncogenic activity at the transcriptional level. The overexpression of the *HMGA1* gene has been described in different subtypes of human pituitary adenomas [40]. *HMGA2* overexpression was also found in non-functional and PRL-secreting pituitary adenomas [40]. It has been reported that HMGA1 and HMGA2 nuclear expression levels are significantly higher in invasive adenomas than in non-invasive ones, particularly in GH-secreting adenomas, in which HMGA2 expression is higher than that in other PitNETs [41].

Gain-of-function somatic mutations of the *PIK3CA* proto-oncogene have been described in different types of PitNETs, including ACTH-secreting, PRL-secreting, and non-functional PitNETs, implicating the PI3K/Akt signaling pathway in tumorigenesis. These mutations were associated with increased PitNET invasiveness [42,43].

Copy-number variations (CNVs) are structural genome rearrangements and chromosomal region gains or losses. They occur frequently in PitNET, with functioning adenomas displaying more CNVs than non-functioning and silent tumors. Chromosomal instability is higher in lactotroph tumors, and the quantity of altered genome is associated with poorer prognosis [44]. Therefore, the landscape of genomic alterations in PitNET includes a large spectrum of gene mutations and chromosomal abnormalities. Still, the genetic mechanisms underlying tumoral growth and biological behavior are not fully understood.

A summary of the genetic alterations in PitNET subtypes is rendered in Table 3.

#### 2.2.2. Epigenetic Profile

Epigenetic changes include heritable modifications to DNA that regulate gene expression without transforming the DNA sequence. These changes influence the accessibility of affected sites, allowing gene transcription or impairing it. The regulation of gene expression depends on histone modifications, DNA methylation, histone variants, remodeling enzymes, and effector proteins. Dysregulation of epigenetic mechanisms at the chromatin or RNA levels may contribute to tumorigenesis, disease progression, and invasiveness.

DNA methylation is a key regulator of gene expression and protein synthesis. It involves adding methyl groups to the DNA molecule mediated by DNA methyltransferases (DNMTs). When located in a gene promoter, DNA methylation typically represses gene transcription. Hypomethylation maintains open DNA strands, allowing transcription. Hypermethylation results in closed DNA conformation, suppressing transcription [45]. Methylation markers could be identified based on ctDNA, the main advantage being that methylation changes are more common among ctDNA fragments than somatic mutations [46]. However, further studies are needed to clarify the role and relevance of methylome analysis.

DNA hypermethylation of several tumor suppressor genes and DNA repair genes has been reported in pituitary adenomas. Among the CpG methylation enzymes, there are two functional DNA methyltransferases (DNMTs)-DNMT1 and DNMT3A—which are overexpressed and are associated with tumor-aggressive behavior and high-methylation status [47].

*NNAT* (Neuronatin) is an imprinted protein-coding gene thought to be involved in brain and pituitary development and maturation and, perhaps, tumor development. It is one of the most abundant transcripts in the pituitary. A hypermethylation-associated loss of *NNAT* has been found in 70% of PitNET, independent of its subtype. This gene and its protein product likely inhibit cellular proliferation within the pituitary [48].

The proteins H-cadherin (coded by the *CDH13* gene) and E-cadherin (coded by the *CDH1* gene) play a key role in cell–cell adhesion. Reduced expression of these proteins by promoter hypermethylation of *CDH13* and *CDH1*, alone or in combination, was detected in PitNET in about half of pituitary adenomas. Loss of these adhesion molecules is associated with tumor aggressiveness. [49].

DAPK (death-associated protein kinase) has a tumor-suppressive function and is an essential regulator of apoptosis and autophagy. Loss of DAP kinase expression has been reported in pituitary tumors. It has been associated with more biologically aggressive tumors than their non-invasive counterparts. Two mutually exclusive mechanisms have been suggested for the silencing of the gene: methylation of the CpG or homozygous deletion of the CpG island [50].

Genome-wide methylation profiling may help identify aggressive and metastatic PitNETs that present with benign histology and without increased proliferation at the time of the first surgery. Jotanovic et al. performed a genome-wide methylation analysis, including copy-number variation (CNV) calculations, on tumor tissue specimens from a large international cohort of 64 patients with aggressive (48) and metastatic (16) pituitary tumors. Three separate clusters were identified: aggressive, metastatic, and benign PitNETs. Thus, DNA methylation analysis could be a valuable tool for the early identification of patients at risk of developing aggressive and metastatic PitNETs [51].

In the somatotroph PitNET, various methylated genes are identified. *GADD45g* (growth arrest and DNA damage-inducible 45) is a tumor suppressor gene implicated in cell cycle arrest, DNA repair, and apoptosis. His expression is lost in the majority of GH-secreting and PRL-secreting pituitary tumors [52].

Galectin-3 (encoded by *LGALS3*) is a protein functioning as an oncogene, with various functions, particularly cell migration, cell adhesion, cell-to-cell interactions, and inhibition of apoptosis. Methylation of the *LGALS3* gene is one mechanism regulating the expression of Gal-3 in pituitary tumors [53].

Aberrant promoter methylation of the *RASSF1A* (human Ras-association domain family 1A) gene results in silencing this tumor suppressor gene in 50% of all subtypes of PitNETs, but is higher in the aggressive ones and potentially correlated with Ki-67 expression [27,54].

In the corticotroph PitNET, the *POMC* gene has been studied. It is a protein-coding gene that encodes a preproprotein that undergoes tissue-specific post-translational processing, resulting in several biologically active peptides, including ACTH. A second promoter of the *POMC* gene was identified. The second promoter is partially demethylated in normal pituitary tissue, highly methylated in silent corticotroph adenomas, and highly demethylated in pituitary and ectopic ACTH-secreting tumors. In contrast, the first promoter is demethylated in all *POMC*-expressing cells and is highly demethylated only in pituitary ACTH-secreting tumors harboring the *USP8* mutation [55].

FGF (Fibroblast Growth Factor) signaling is critical in pituitary development. Promoter methylation epigenetically silences the *FGFR2* (Fibroblast Growth Factor Receptor 2) gene in pituitary tumors. The melanoma-associated antigen-3 (*MAGE-3*) is a target of FGFR2. His promoter is hypomethylated in pituitary tumors. It is a putative oncogene responsible for p53 dysregulation in PitNET. The studies show that *MAGE-3* is upregulated, whereas *FCFR2* is downregulated. Therefore, while FCFR2 plays a growth-inhibitory tumor-suppressive role, *MAGE-3* is considered to have growth-promoting oncogenic functions [56]. The expression of *FGFR4* also correlates with tumor invasiveness.

TSP-1 (Thrombospondin-1) is a matricellular glycoprotein involved in cell-to-cell and cell-to-matrix interactions. It is associated with platelet aggregation, angiogenesis, and tumorigenesis. Marked downregulation of the *TSP-1* encoding gene was identified in corticotroph PitNET.

The tumor suppressor gene *CDKN2A* (cyclin-dependent kinase inhibitor 2A) encodes proteins (mainly p16 and p14) that regulate two critical cell cycle regulatory pathways: the p53 pathway and the RB1 pathway. The mechanism of downregulation by methylation of promoter sequences was detected in most gonadotroph, lactotroph, plurihormonal, and null cell adenomas (36 of 44, 82%), but it was rare in somatotroph (1 of 13 cases, 8%) and corticotroph adenomas (1 of 15 cases, 7%) [57]. This is the most common mechanism of *CDKN2A* inactivation in PitNET. It is probably not an initial event but is acquired during adenoma progression. The p16 protein inactivates the function of CDK–cyclin complexes, inhibiting the retinoblastoma protein (pRb), which controls the cell cycle’s G1-S phase checkpoint. Downregulating *CDKN2A* results in a reduced expression of p16. The retinoblastoma protein becomes phosphorylated, enabling cell cycle progression and increasing proliferation.

*MEG3* (Maternal Imprinted Gene 3) is an imprinted tumor suppressor gene modified in PitNET via DNA methylation. Hypermethylation of the *MEG3* regulatory region is associated with the loss of *MEG3* expression in non-functioning pituitary tumors. Loss of MEG3 leads to decreased expression of p53, increased cell survival through activated cell cycle progression, and decreased apoptosis [58].

*ENC*1 (Ectodermal-Neural Cortex 1) is a protein-coding gene. Methylated *ENC1* has been found in non-functioning PitNET, and expression levels are reportedly lower in invasive adenomas than in non-invasive ones [59].

The DNA methylation and expression levels of *FAM90A1* (Family with Sequence Similarity 90 Member A1) and *ING2* (Inhibitor of Growth Family Member 2) are associated with tumor regrowth in non-functioning PitNETs and may serve as biomarkers for predicting the prognosis in these cases [60]. The *ING2* gene belongs to the family of tumor suppressor genes. It regulates the activity of histone acetyltransferase and histone deacetylase complexes and plays a role in DNA repair, proliferation, and apoptosis.

A summary of the epigenetic alterations in PitNETs and their clinicopathological significance is rendered in Table 4.

### 2.3. Cell-Free RNA (cfRNA)

Cell-free RNA (cfRNA) consists of RNA molecules released from non-tumoral and tumoral cells in bodily fluids through apoptosis, necrosis, and active secretion. CfRNAs are encapsulated within extracellular vesicles or form complexes with proteins or lipoproteins that protect them from the activity of endogenous ribonucleases (RNases), maintaining their stability in serum, plasma, and other bodily fluids. These molecules offer tumor-specific signatures and have high stability in circulation and thus can be used as liquid biopsy-derived biomarkers to evaluate tumor pathophysiological changes.

According to the central dogma of molecular biology, information is passed from DNA (gene) to protein through messenger RNA (mRNA), a coding RNA [61]. It has been found that two-thirds of the human genome is transcribed, but only 2–5% of the transcribed genome is translated into proteins [62]. The remaining encodes a wide range of non-coding RNA molecules that control various cellular processes and play critical roles in many pathological conditions, including tumors. The binary classification of coding versus non-coding is now outdated because RNAs with both coding and non-coding functions were discovered, which were considered bifunctional RNAs. Moreover, the same non-coding RNA can act as an oncogene in some cancer types and a tumor suppressor in others [62]. This entangled field has just started to be explored. Several subtypes of cfRNA have been associated with PitNETs, mainly non-coding regulatory RNAs like microRNA (miRNA), long non-coding RNA (lncRNA), and circular RNA (circRNA). There are multiple interactions between miRNA and lncRNA/circRNA, especially the competing endogenous RNA (ceRNA) mechanism, which plays an essential role in the pathogenesis of PitNET and its subtypes [63]. According to this mechanism, transcripts such as lncRNA and circRNA competitively bind to miRNAs, thus forming a complex regulatory network to realize certain functions.

The integration of the non-coding RNAs in the clinic as reliable, noninvasive biomarkers is far from being achieved. Still, it is essential to be informed about the extensive research within the complex field of regulatory RNAs, which is progressively unraveling.

#### 2.3.1. MicroRNA (miRNA)

Micro ribonucleic acids are small non-coding RNAs with a length of 19–25 nucleotides, involved in mRNA silencing and the post-transcriptional regulation of gene expression. They control about 50–60% of coding genes [18,29]. The overexpression of miRNAs targeting tumor suppression genes and suppressed expression of miRNAs that target oncogenes are implicated in tumorigenesis and progression [64]. They are key players in the modulation of critical growth regulatory pathways in cancer pathogenesis. Tumor-associated miRNAs are generally classified into one of two subcategories: tumor suppressor miRNAs (tumor-suppressor-miRs) and oncogenic miRNAs (onco-miRs) [65]. Interestingly, several miRNAs appear to possess dual functionality, acting as both tumor suppressors and oncogenes. These functions manifest in a context-dependent manner and relate to specific stages of cancer [62,65]. The expression patterns of miRNAs exhibit high specificity for both the tissue and cell of origin, and there are definite altered miRNA signatures in various cancer types [66,67]. In addition, the concentration of miRNAs in blood circulation is higher than that of other types of cfRNAs, and they have the advantages of high stability, not only in the bloodstream but also in deleterious conditions, such as acidic or alkaline pH, high temperature, and multiple freeze–thaw cycles [64]. Thus, miRNAs could be a liquid biopsy-based biomarker for the diagnosis and prognosis of cancer.

When it comes to PitNETs, it is well known that in most cases, these tumors originate from a single cell, while very rare multiple clonalities have been observed [68]. However, although the complete process of tumor formation is yet to be fully uncovered, it has been demonstrated that miRNA has a key role in tumorigenesis. One of the first discoveries regarding the involvement of miRNA in PitNETs regards miR-15a and miR-16-1 [69]. These nucleic acids are located on a chromosomal part, which is mainly deleted in PitNETs, and are expressed at lower levels than healthy hypophyses. While their expression was inversely correlated to tumoral diameter, it has been concluded that the downregulation of these types of miRNA in PitNETs is associated with tumoral growth [69]. The last decades have shed more light on the roles and implications of nucleic acids in pituitary lesions. MicroRNAs have different roles depending on the type of PitNET secretion.

Multiple correlations have been demonstrated between miRNAs and somatotropinomas. In these types of tumors, the focus lies on the excessive secretion of growth hormone (GH) and Insulin-like growth factor 1 (IGF1), leading to acromegaly. Nevertheless, this mechanism alone cannot completely explain the occurrence of acromegaly. Thus, further research is needed [70]. However, acral enlargement is the main symptom, particularly bone enlargement, and this is the reasoning behind a functional association between tumoral cells and osteoblasts. New research shows that exosomal miR-21 downregulates the expression of programmed cell death protein 4 (PDCS4) and Smad7 to promote osteoblast proliferation and bone formation [71]. Thus, besides hormonal hypersecretion, the communication between tumoral cells and miRNA is considered a novel mechanism unveiled in acromegaly. More data suggest that exosomes originating from somatotropinomas promote osteoblast proliferation through miR-21/PDCD4/AP-1 and Smad7/Runx2 pathways [71]. The miR-21-5p, found in plasma exosomes, regulates apoptotic pathways and cellular proliferation, and it has the potential to be a non-invasive marker for disease progression and therapeutic response, especially in patients undergoing chemotherapy or radiotherapy [71].

Belaya et al. also studied the link between acromegaly and miRNA expression patterns. Their research demonstrated that miRNA expression is involved in mesenchymal stem cell commitment, which, along with the downregulation of *TWIST1* expression, shows the negative effect of acromegaly on osteoblastogenesis [72].

Korkmaz et al. studied the expression levels of miR-29c-3p in patients with acromegaly [73]. The authors discovered that significantly lower levels of this miRNA type were associated with inadequately controlled acromegaly, while the risk of developing this condition was higher when miR-29c-3p was downregulated [73].

Zhao et al. studied the exosome miRNA expression profiling in somatotropinomas and discovered lower expression of hsa-miR-320a and hsa-miR-423-5p [74]. While further testing the exact function of these nucleic acids, the authors showed that miR-423-5p inhibited cellular proliferation, induced apoptosis, and reduced GH release and migration of GH3 cells [74].

It is worth mentioning that the miRNA is also involved in AIP-mutated somatotropinomas. These mutated pituitary tumors overexpress miR-34a, which, in turn, downregulates Gαi2 expression, increases cAMP concentration, and promotes cellular growth. Furthermore, not only does the upregulation of this miRNA affect the hormonal and antiproliferative response to octreotide, but it is also a downstream target of mutant *AIP* that promotes a cellular phenotype reflecting the aggressive clinical characteristics observed in AIPmut+ acromegaly [75]. These findings are consistent with previously published ones that showed a negative impact on treatment efficacy in AIPmut + PitNETs [76].

The miRNAs have been evaluated in patients with prolactinomas. The miR-137, a proven tumor suppressor in melanomas and gliomas, is downregulated in lactotroph PitNETs, especially in invasive prolactinomas, as it acts as an inhibitor of microphthalmia-associated transcription factor (MITF) and is involved in cell differentiation, proliferation, and survival [77]. MITF is mainly implicated in the differentiation of certain types of cells, such as melanocytes, osteoclasts, and mast cells. Still, the upregulation of this transcription factor is demonstrated in prolactinomas, particularly in the invasive ones [77]. Furthermore, miR-137 could act as a tumor suppressor by inhibiting the Wnt/β-catenin signaling pathway and suppressing cell proliferation, given that it causes the downregulation of MITF and the destabilization and inhibition of nuclear translocation of β-catenin. Moreover, it also determines the upregulation of WIF-1, a key inhibiting factor that inactivates the Wnt signaling pathway [77]. Further analysis showed that NOVA1, DTL, and RAB27B were targeted by miR-99a-3p, inhibiting cellular growth in prolactinomas [78].

The miR-93 has also been proven to be involved in the pathogenesis of prolactinomas. This miRNA mediates resistance to dopamine agonists like cabergoline by targeting Autophagy Related 7 (ATG7), which complicates the treatment of certain PitNETs. ATG7 is an essential protein for cellular autophagy. The miR-93 contributes to dopamine agonist resistance in prolactinomas by inhibiting autophagy-related pathways, such as the expression of ATG7, which is needed for autophagic degradation processes. Autophagy is an essential process for maintaining cellular homeostasis and has been implicated in resistance to temozolomide (TMZ) in glioblastoma. By regulating autophagy, miR-93 influences TMZ response in these tumors. Suppressing miR-93 in PitNETs could restore autophagic pathways, enhancing TMZ sensitivity and improving treatment outcomes for prolactinomas and potentially other PitNET subtypes. The miR-9 promotes EMT, a key process that stimulates cell migration and invasion. This suggests that miR-9 may contribute to the aggressive behavior of PitNETs, especially in those with invasive characteristics [79,80].

A recent study on 41 consecutive patients with ACTH-dependent Cushing’s disease showed that miRNAs are differentially expressed in these patients versus healthy individuals. Furthermore, the plasma miRNA levels differed between patients with Cushing’s disease and patients with ectopic ACTH secretion. The exact types of miRNA that showed promising results as biomarkers for differentiating these patients were miR-16-5p, miR-145-5p, and miR-7g-5p [81]. The miR-145-5p has been associated with invasive features of PitNETs, particularly in ACTH-secreting tumors, and showed differences between ACTH-producing pituitary PitNETs and ectopic ACTH secretion syndromes. The miR-122-5p, miR-141-3p, and miR-375 served as circulating indicators of corticotroph tumor behavior, with distinct levels correlated to treatment, specifically reductions in ACTH levels postoperatively [81].

Although many mechanisms behind Cushing’s disease have been studied, its complete pathogenesis, as caused by PitNETs, is yet to be understood. New research suggests that secreted angioinhibitory factor thrombospondin-1 (TSP-1) expression is significantly lower in patients with corticotropinomas versus healthy individuals, and is a direct target of miR-449c [82]. TSP-1 mediates cell attachment, glycosaminoglycan binding, angiogenesis inhibition, Transforming Growth Factor beta (TGF-β), and inhibition of matrix metalloproteinases. Furthermore, the overexpression of this adhesive glycoprotein in corticotropinomas suppressed cellular proliferation, migration, and invasion, while the activity of miR-499c stimulated tumorigenesis by directly inhibiting the expression of TSP-1. In a similar manner, additional research suggests that low expression of lncTHBS1 and TSP-1 is correlated with high expression of miR-449c in patients suffering from Cushing’s disease, and lncTHBS1 could suppress the development of this disease [82].

The detection of circulating miRNAs, such as miR-22-3p, miR-27a-3p, and miR-320b, has shown a correlation with cortisol levels, which can provide valuable insights into PitNET function, particularly in the context of the low-dose dexamethasone test [83].

In the last decade, various studies have focused on the role of miRNAs in the pathology of gonadotrophinomas. Differentially expressed genes were shown to be regulated by miR-374, miR-153, miR-145, and miR-33, thus contributing to the tumoral development in preclinical studies [84]. Moreover, the upregulated differentially expressed genes were particularly enriched in the neuroactive ligand–receptor interaction, whereas the downregulated differentially expressed genes were enriched in cell cycle [84]. In glioblastoma, miR-145 has been shown to enhance Temozolomide sensitivity by targeting A Disintegrin and Metalloprotease 17 (ADAM17), which influences apoptosis and cell proliferation [85]. Restoring miR-145 expression in PitNETs may reduce tumor invasiveness and progression, thus sensitizing the tumors to TMZ. This makes miR-145 a promising candidate for improving treatment responses in PitNETs, particularly in cases where the tumors are resistant to other therapies [86].

The miR-143-3p has also been identified in the plasma of patients with gonadotropinomas. It has been stated that this type of miRNA regulates signaling pathways related to tumorigenesis and could serve as both diagnostic and prognostic biomarkers for aggressive gonadotropinomas, offering insights into its behavior and treatment response [87]. Nemeth et al. concluded that plasma miR-143-3p levels decreased in patients with gonadotropinomas, indicating successful surgery. These conclusions were drawn after observing that miR-143-3p was downregulated in late postoperative but not in early postoperative plasma samples compared with preoperative ones, exclusively in gonadotropinomas [88].

Slight differences in miRNA expression profiles were discovered in invasive versus noninvasive PitNETs, and six types of miRNAs were concluded as biomarkers for invasiveness: Hsa-miR-184, hsa-miR-181a-2-3p, hsa-miR-93-3p, hsa-miR-574-5p, hsa-miR-185-5p, and hsa-miR-3200-5p [89].

In invasive non-functional PitNETs, miR-185-5p is upregulated, though its reliability as a biomarker for invasiveness is still being evaluated [89].

The miR-137 is downregulated in invasive non-functioning PitNETs. This nucleic acid regulates the Wnt signaling pathway by targeting Wnt Inhibitory Factor 1 (WIF1), which binds Wnt proteins and prevents them from triggering signaling. The ectopic expression of miR-137 inhibited these tumors’ proliferative and invasive features, whereas its suppression promoted the proliferation and invasion of neoplastic cells [90].

Elston et al. demonstrated that the Wnt pathway target gene cyclin D1 is upregulated in non-functioning PitNETs, and by transfecting GH3 cells with WIF1, a decrease in cellular proliferation was observed. The authors concluded that WIF1 could act as a tumor suppressor, particularly in non-functioning lesions, while Wnt pathways have a significant role in tumorigenesis [91]. In a similar manner, Song et al. suggested that aberrant regulation of the Wnt signaling pathway is a key factor in tumorigenesis. The authors also discovered that the overexpression of miRNA-137 inhibits cellular proliferation and invasion, and the WIF1 level was upregulated after the overexpression of miRNA-137. These findings revealed that miRNA-137 has a crucial role in the Wnt pathway by affecting promoter methylation of *WIF1* [92].

The miR-370-3p is also downregulated in high-grade non-functioning PitNETs, where it regulates cell proliferation and invasiveness by targeting HMGA2, a protein that may act as a transcriptional regulating factor [64].

Additionally, miR-145-5p, which is downregulated in non-functioning PitNETs, contributes to invasiveness and cell proliferation by targeting Tumor Protein Translationally Controlled 1 (TPT1), thought to promote cell survival by enhancing the anti-apoptotic response and suppressing pro-apoptotic activities. The circOMA1, a type of circRNA, is involved in the dysregulation of miR-145-5p by acting as a sponge and promoting proliferation and invasion of the non-functioning pituitary tumor cells [93].

It has been stated that miR-146b is associated with poor prognosis and tumor invasiveness. While it plays a central role in regulating immune responses and inflammation, it suppresses tumor invasiveness and metastasis. Monitoring miR-146b could provide valuable insights into the likelihood of recurrence and tumor progression [94].

The miR-141, a member of the miR-200 family, regulates epithelial–mesenchymal transition. Elevated levels of miR-141 are observed in more aggressive PitNETs. Its increased expression can help differentiate between aggressive and non-aggressive PitNETs, aiding in early diagnosis, particularly when traditional imaging methods may not provide clear results. Given its role in epithelial–mesenchymal transition, miR-141 can serve as a prognostic marker to assess tumor aggressiveness and predict the risk of recurrence following treatment [95].

Hypoxic conditions induce the miR-210 and regulate pathways that support tumor growth and survival in low-oxygen environments, contributing to tumor aggressiveness [69].

The miR-34a is downregulated in PitNETs, which facilitates tumor growth and survival. Its reduced expression is linked to aggressive tumor behavior, as it targets pathways such as Notch1, Notch2, cyclin-dependent protein kinase-6 (CDK6), and proteins involved in Akt/mTOR and Wnt signaling [96,97].

The miR-30c is associated with cell proliferation, migration, and invasion, which suggests its role in tumor progression [95]. The miR-222, a marker of cell cycle regulation, is often overexpressed in PitNETs, correlating with increased tumor growth and poor prognosis [98].

The miR-155 targets HMG-box transcription factor 1 (HBP-1), Mitogen-Activated Protein Kinase 13 (MAPK13), and Mitogen-Activated Protein Kinase 14 (MAPK14). MAP kinases act as an integration point and are involved in proliferation and differentiation. The miR-155 also promotes activation of the Wnt/β-catenin signaling pathway, contributing to tumor progression [99,100].

The miR-200a is elevated in invasive PitNET cases, representing a marker for tumor invasiveness [101].

Finally, miR-543 is upregulated in PitNETs and promotes tumor proliferation and invasion by targeting Smad7. This protein is a TGF-β type 1 receptor antagonist that inhibits TGF-β signaling. Enhanced TGF-β signaling enables cancer cell invasion and dissemination, stem cell properties, and therapeutic resistance [64,102].

#### 2.3.2. Long Non-Coding RNA (lncRNA)

Long non-coding RNAs (lncRNAs) are a heterogeneous class of RNA molecules longer than 200 nucleotides. Even though they do not encode proteins, these transcripts play a critical role in multiple cellular processes, influencing cell identity and functions. They are involved in chromatin organization, gene transcription, post-transcriptional regulation, post-translational effects, and intracellular trafficking. The human genome encodes more than 10,000 lncRNAs, but only a few lncRNAs are currently characterized. Various lncRNAs have been associated with different cancers, but our knowledge is still limited. In 2000, Hanahan and Weinberg proposed six hallmarks of malignant transformations that define cancer, including (1) self-sustaining proliferative signaling, (2) evasion of growth inhibition, (3) avoidance of apoptosis (normal, programmed cell death), (4) uncontrolled proliferation, (5) angiogenesis and (6) tissue invasion and metastasis [103,104,105]. Long non-coding RNAs are involved in all these mechanisms. They can interfere with chromatin remodeling and, thus, with DNA packaging, gene expression, and DNA replication. LncRNAs also function as ceRNAs during tumorigenesis. They can interact with miRNAs to act as sponges to downregulate their effects and thus have a regulatory function on post-transcriptional modifications. LncRNAs can interact with transcription factors to activate or repress gene expression or get involved in splicing regulation. They can also determine post-translational protein modifications. It has been demonstrated that lncRNAs exhibit tissue-specific expression and have a relatively stable secondary structure in serum, plasma, and other bodily fluids. Some lncRNAs are selectively loaded into exosomes, which protect them from endogenous ribonucleases. Therefore, lncRNAs can be suitable biomarkers for diseases, including PitNETs.

Several lncRNAs have been identified in different tissue samples and are now established molecular markers of tumorigenesis in PitNETs. However, their expression in liquid biopsy has not been systematically evaluated to date. We provide a working list of lncRNAs that may be functionally relevant to PitNET tumorigenesis and can be investigated in body fluids to find their potential as biomarkers in these tumors.

Xing et al. reported the first genome-wide profiling of lncRNAs, identified mRNAs differentially expressed in non-functioning PitNET using tissue specimens, and attempted to construct a lncRNA-mRNA co-expression network. The authors found 113 lncRNAs and 80 mRNAs differentially expressed in non-functioning pituitary adenomas (NFPA) using microarray analyses and further confirmed differential expression in NFPA for ten of the 113 lncRNAs using real-time quantitative reverse transcription polymerase chain reaction (qRT-PCR) [106]. Some of these ten doubly confirmed lncRNAs have already been established as linked with malignant transformations, like Maternally Expressed Gene 3 (MEG3) and ENST00000501583. MEG3 functions as a critical tumor-suppressor lncRNA via p53-dependent and p53-independent apoptotic pathways. His expression is downregulated in both non-functional and invasive PitNETs. ENST00000501583 expression is also downregulated in NF-PitNET. The study also identified n334366 as the most significantly downregulated lncRNA in NF-PitNET. It was revealed that it is positively correlated with DLK1 (an mRNA regulated by the Notch pathway, which has been implicated in NFPA tumorigenesis) [106]. His function has not yet been elucidated. The downregulation of the *MEG3* expression (the imprinted gene that encodes a specific lncRNA) in some pituitary tumor subtypes, mainly in clinically nonfunctioning adenomas, was confirmed by several studies, and overall hypermethylation in the important regulatory regions of the gene is an additional mechanism that contributes to the specific loss of expression [107,108,109,110]. The restoration of *MEG3* expression through demethylating agents has been proposed as a potential therapeutic strategy, focusing on the reactivation of its tumor-suppressing effects.

The expression of the long non-coding RNAs MEG3, HOTAIR (Hox transcript antisense intergenic RNA), and MALAT-1 (Metastasis-associated Lung Adenocarcinoma Transcript 1) was investigated in non-functioning PitNET development and invasion [110]. HOTAIR is involved in chromatin remodeling processes, contributing to tumor invasiveness and metastasis. MALAT1 has been linked to accelerated tumor progression and poor prognosis in various tumors. The study demonstrated decreased MEG3 expression and increased HOTAIR expression in NF-PitNET, with dysfunctions accentuated in invasive NF-PitNET. There was no significant association between MALAT-1 expression and tumor characteristics [110,111]. Thus, MEG3 and HOTAIR lncRNAs are potential biomarkers for NF-PitNET and could be associated with aggressive tumor behavior [110].

H19 is a multifunctional lncRNA involved in embryo development and growth, glucose metabolism, and tumor development. It has a complex role and plays opposite roles in different tumors. His expression is downregulated in PitNET and is correlated with tumor progression, suggesting that H19 acts as a tumor suppressor. Wu et al. demonstrated that H19 suppresses pituitary tumor cell proliferation and tumor growth through the H19/mTORC1/4E-BP1 axis by blocking 4E-BP1 phosphorylation and function, but not affecting mTORC1 complex integrity [112]. Dopamine agonists (DAs), such as bromocriptine and cabergoline, are the first-line treatment for PRL-PitNET, but 10-20% of the patients still fail to respond to these medical therapies. Cabergoline inhibits pituitary cell proliferation and mediates autophagic cell death. Interestingly, H19-induced tumor suppression is superior to cabergoline treatment. Also, H19 promotes the tumor’s sensitivity to DAs by functioning as a sponge for miR-93, inhibiting its expression and increasing the expression of ATG7, a protein involved in autophagy [80]. Notably, plasma exosome-derived H19 is significantly downregulated in PitNET. Therefore, H19 can be not only a potential biomarker of PitNET but also a potential therapeutic target, implicated in counteracting drug resistance, mainly in prolactinomas [112,113]. Lu et al. reported remarkably higher H19 expression in invasive GH-PitNET compared to that in non-invasive ones, suggesting that this lncRNA may play a role in specific tumor invasion [111].

RPSAP52 (Ribosomal Protein SA Pseudogene 52) is an antisense lncRNA for the *HMGA2* gene, with an overexpression that is critical in developing pituitary adenomas. RPSAP52 is overexpressed in gonadotroph-secreting and PRL-secreting PitNET and shows a variable behavior in GH-secreting PitNET. RPSAP52 enhances HMGA2 protein expression in a ceRNA (competing endogenous RNA) dependent way, acting as a sponge for miR-15a, miR-15b, and miR-16, which have already been described to be able to target HMGA2 [114]. It also positively modulates HMGA1 and promotes cell growth by enhancing the G1-S transition of the cell cycle.

LncRNA clarin 1 antisense RNA 1 (CLRN1-AS1) was found to be downregulated in PRL-secreting PitNET tissue samples. It can suppress cell proliferation, promote apoptosis, and inhibit autophagy, having a tumor-suppressor property. Forkhead box P1 (FOXP1) was verified to be a transcription suppressor of CLRN1-AS1. CLRN1-AS1 acts as a sponge of miR-217 to upregulate the dickkopf WNT signaling pathway inhibitor 1 (DKK1). DKK1 is an inhibitor of the Wnt/β-catenin signaling pathway, a crucial pathway in the progression of tumors. Hence, the overexpression of DKK1 inactivates the Wnt/β-catenin signaling pathway [115]. Analyzing the upstream molecular mechanism of CLRN1-AS1, Forkhead box P1 (FOXP1) was identified as a transcription suppressor [115].

In ACTH-secreting PitNET, lncTHBS1 has low expression, along with low expression of TSP-1 (Thrombospondin-1), a matricellular protein involved in cell-to-cell and cell-to-matrix interactions and associated with platelet aggregation, angiogenesis, and tumorigenesis. LncTHBS1 functions as a ceRNA with miR-449c and induces a downregulation of the *TSP-1* gene, which encodes TSP-1, an inhibitor of proliferation in endothelial cells, which induces apoptosis, and suppresses the cell cycle [82]. Therefore, lncTHBS1 is a tumor-suppressor RNA.

PVT1 (Plasmacytoma Variant Translocation 1) and NEAT1 (Nuclear Paraspeckle Assembly Transcript 1) are lncRNAs that regulate cell proliferation, apoptosis, differentiation, and cell cycle transition. PVT1 has an oncogenic role by mediating the regulation of the Wnt/β-catenin signaling pathway, and NEAT1 acts as a miR-148b-3p sponge, thus affecting the proliferation and invasion of tumor cells. In PitNEt, an upregulation of PVT1 and NEAT1 was reported [116].

CCAT2 (colon cancer-associated transcript 2) is an lncRNA reported as significantly upregulated in PitNET, contributing to carcinogenesis and progression. CCAT2 overexpression in human pituitary adenoma cells occurs partly due to the transcription factor E2F1 binding to its promoter region and promoting transcription. CCAT2 significantly increases the PTTG1 protein level, suggesting that CCAT2 influences the stability of PTTG1 and suppresses its degradation. PTTG1 has been identified as a proto-oncogene. Therefore, CCAT2 exerts an oncogenic function in pituitary adenomas [117].

ANRIL (Antisense Non-coding RNA in the INK4 Locus) is another lncRNA involved in cell cycle control and proliferation [118]. In a study aiming to investigate the specific profiles of ANRIL and miR-200a PitNET, Beylerly et al. analyzed plasma and tissue samples from invasive and non-invasive tumors. The expression levels of ANRIL and miR-200a were significantly increased in tissue samples and plasma during the preoperative period in patients with invasive PitNET compared to patients with non-invasive PitNET. Interestingly, the post-operative analysis revealed decreased circulating plasma levels of ANRIL and miR-200a in patients with invasive PitNETs [119]. These findings suggest that ANRIL may play a role in tumor invasiveness and could serve as a valuable marker in liquid biopsy for invasive PitNETs and for evaluating disease progression.

LINC00473 was identified as the most upregulated lncRNA in invasive PitNET. Its overexpression in these tumors restricts miR-502-3p through the ceRNA mechanism, and upregulates *KMT5A* expression, which encodes KMT5A, a histone methyltransferase. The upregulation of KMT5A increases the expression of cyclin D1 and CDK2, key proteins of the cell cycle, which favors cell cycle progression and promotes cell proliferation, leading to tumor proliferation and invasion [120].

Long non-coding RNA TUG1 (taurine upregulated gene 1) was demonstrated to be upregulated in invasive PitNET. TUG1 sponges miR-187-3p and promotes TESC (tescalcin) overexpression and implicit activation of the NF-κB (nuclear factor-kappa B) signaling pathway, which promotes tumorigenesis [121].

LncRNA IFNG-AS1 is overexpressed in PitNET tissue samples, and this upregulation was linked to invasiveness. Its oncogenic role depends on interacting with ESRP2 (epithelial splicing regulatory protein 2), which serves as an epithelial-specific regulatory factor in cells and may be involved in alternative mRNA splicing, which is critical for tumor progression and the epithelial–mesenchymal transition [122].

C5orf66-AS1 (also known as Epist and CTC-276P9.1) is verified to be underexpressed in null cell adenomas than in normal pituitary tissues and in invasive adenomas than in non-invasive adenomas. These results indicate its role as a suppressor of tumor development and invasion [123].

#### 2.3.3. Circular RNA (circRNA)

Circular RNAs (circRNAs) are single-stranded RNA molecules with a covalently closed circular structure, which protects them from RNAase cleavage. Therefore, they can be stable in peripheral blood and other body fluids. However, these molecules can also be transported by exosomes, where they retain their biological activity, which can be transferred to recipient cells [124]. They proved a cell-type and tissue-specific expression and tend to accumulate in cells with a low proliferation rate, such as neurons [125,126]. Consequently, their characteristics make circRNAs promising biomarkers for liquid biopsy research. CircRNAs are mainly generated by back-splicing of sequences within pre-mRNA molecules. They are associated with critical biological functions, including regulating gene expression through epigenetic or genomic effects, or interacting with miRNAs or proteins [124,125]. While they can modulate gene expression at both transcription and splicing levels, several circRNAs act as miRNA sponges, reducing miRNA levels and mediating their target genes’ regulatory potential. Furthermore, they can interact with proteins, activating or inhibiting their functions. A small proportion can be translated into functional peptides. Although it has been reported that circRNAs are important molecular modulators in cancer initiation and progression, many questions remain to be explored [125]. Recent studies indicated the central involvement of circRNAs in the development and progression of several diseases and neoplasms, including PitNETs [63].

In somatotropinomas, 1938 circRNAs are upregulated, while 1601 are downregulated, in comparison to healthy individuals [127]. The most specific upregulated circRNA in these tumors was hsa_circ_0001368, which was also correlated to invasiveness and the serum level of GH. Moreover, its knockdown inhibited cellular proliferation, invasion, and hypersecretion of GH, with a positive correlation with the pituitary-specific transcription factor Pit-1. Taken together, these findings suggest that the identified circRNA might be a promising potential biomarker and therapeutic target in somatotropinomas [127].

As already mentioned in the section regarding miRNA and non-functioning PitNETs, circRNA and miR-145-5p are both key elements in the pathogenesis of these lesions, as the circOMA1, a type of circRNA, is involved in the dysregulation of miR-145-5p by acting as a sponge and promoting the proliferation and invasion of the non-functioning pituitary tumor cells [93].

Zhang et al. performed in vivo and in vitro analyses of distinctive circRNA patterns in non-functioning PitNETs and observed significantly different profiles in these tumors versus healthy individuals [128]. The authors demonstrated that circVPS13C is significantly upregulated in these tumors, while silencing it leads to the overexpression of IFITM1. Consequently, the increased expression of IFITM1 reverses the biological effects of circVPS13C. In clinical settings, the expression of this circRNA is markedly higher in high-risk non-functioning PitNETs. Moreover, the study concluded that, 7 days post-transsphenoidal excision, this circRNA is downregulated in the patient’s serum. Overall, these findings indicate that circVPS13C is a critical regulator in the tumorigenesis and proliferation of non-functioning PitNETs [128].

In like manner, Guo et al. studied the link between circRNAs and non-functioning PitNETs and discovered a two-circRNA signature that can predict recurrence and/or progression. These signatures are represented by hsa_circ_0000066 and hsa_circ_0069707, and their discriminative power was greater compared to age [129].

Wang et al. studied the expression profile of circRNAs in 75 non-functioning PitNETs, of which 65 were invasive. While the results showed that the expression of hsa_circ_0058735, hsa_circ_0066025, and hsa_circ_0063523 was not statistically significant between the two groups, they also showed that there might be an important correlation between hsa-miR-1184, hsa-miR-942, and hsa-miR-1208 and tumor invasiveness [130].

Another example of circRNA is circ-ABCB10, which has been found to promote cellular proliferation and migration. Its expression correlates with increased tumor aggressiveness, suggesting that it may play a role in the progression of PitNETs. Its elevated levels could serve as an indicator of aggressive tumor behavior, making it a potential target for further investigation as a biomarker for PitNET malignancy [131].

The hsa_circRNA_102597 is downregulated in invasive non-functioning PitNETs, suggesting a possible role in suppressing tumor progression. This is associated with tumor grade and diameter, further supporting its involvement in regulating the aggressive characteristics of PitNETs. The downregulation of hsa_circRNA_102597 in invasive tumors could potentially be used as a marker for assessing the severity of PitNETs and guiding clinical management [132]. These findings show that circRNAs play a more significant role in non-functioning PitNETs. However, further research is needed to confirm its major involvement in these tumors and discover whether there are implications for other types of PitNETs.

A summary of cfRNAs involved in PitNETs with subtype associations and clinicopathological roles has been rendered in Table 5.

### 2.4. Exosomes

**Figure 2:**
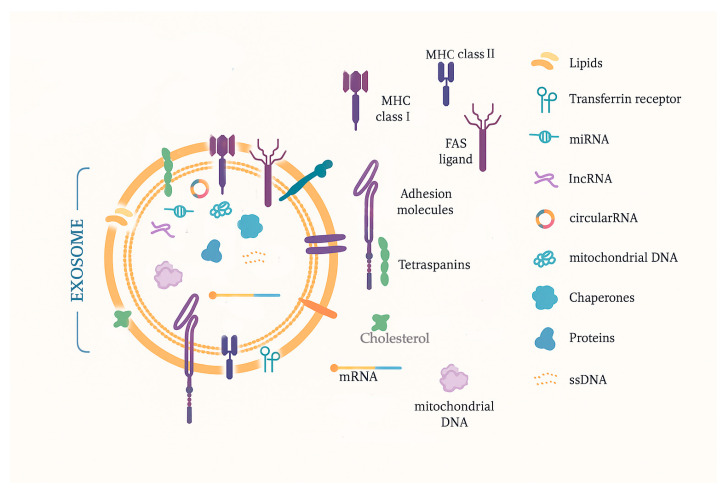
A schematic rendering of an exosome and its components.

Exosomes are extracellular vesicles of nanometric size (30–150 nm) released in a paracrine way, and they can be found in biological fluids, where they are easily accessible. They transport bioactive molecules and are known as mediators of cell-to-cell communication. The lipid double-layer membrane structure offers protection against cargo degradation. They also contain typical markers indicating the cellular source (mainly cell surface receptors encapsulated during endocytosis). Some markers could be not only tumor-specific but could also identify the type of tumor [18]. These characteristics make them a promising natural source of disease biomarkers, which could be identified by liquid biopsy. In clinical oncology, a technical approach to providing quantitative and qualitative data is important because exosome plasmatic levels are higher in tumor patients, and exosome molecular cargo contains informative tumor markers, proteins, lipids, metabolites, and nucleic acids (mRNA, lncRNA, miRNA, circRNA, DNA, etc.). A summarized rendering of the exosomal aspect and components can be observed in Figure 2.

Exosomal molecules are involved in tumor growth, angiogenesis, epithelial–mesenchymal transition, invasiveness, cancer cell immune escape, and drug resistance [133,134]. Therefore, exosomes have great potential as sensitive and precise biomarkers for early cancer detection, disease monitoring, and treatment response evaluation. This can transform personalized therapy.

Exosome-derived mRNA was studied by Yu et al., focusing on circulating biomarkers for screening the invasiveness of non-functioning PitNETs. They used the extraction of exosomal mRNA and detection of mRNA expression of candidate genes related to tumor progression or invasion, such as cyclin-dependent kinase 6 (*CDK6*), ras homolog family member U (*RHOU*), and spire type actin nucleation factor 2 (*SPIRE2*). As a result, *CDK6* and *RHOU* mRNA expressions were upregulated in invasive tumors compared to non-invasive ones. The mRNA levels of *CDK6* and *RHOU* in serum exosomes were significantly positively correlated. Thus, *CDK6* and *RHOU* mRNA in serum exosomes can be used as markers for predicting the invasiveness of NF PitNET, both in clinical screening and postoperative evaluation [135].

Exosome-derived miRNA profiles of non-functioning PitNETs were investigated by Lyu et al. to identify markers for screening and prognosis. They found 18 upregulated and 36 downregulated miRNAs that showed significant expression alterations in NF-PitNET patients. After validation, exosomal hsa-miR-486-5p, hsa-miR-151a-5p, hsa-miR-652-3p_R+1, and hsa-miR-1180-3p were promising biomarkers for the diagnostic and screening of NF-PitNETs, in which miR-486-5p was the most competent one. Prospective follow-up (33 months) reveals that exosomal hsa-miR-486-5p was an efficient predictive biomarker for the progression or recurrence of studied tumors. It might control tumor progression by epigenetic regulation of MAPK signaling pathways [136].

Zhao et al. (2019) studied the serum exosomal miRNA expression signature of somatotroph adenomas to identify a specific biomarker of these tumors. There were 169 known miRNAs differently expressed between somatotroph adenomas and normal samples, including 121 upregulated and 48 downregulated miRNAs. The miR-423-5p had a lower expression level in somatotroph adenomas. *PTTG1* was the target mRNA of miR-423-5p, and a high expression of PTTG1 in GH-secreting PitNET was noted, which was associated with cell proliferation and migration. The results verified the central role of low miR-423-5p in promoting tumorigenesis and PTTG1 as a potential biomarker for somatotroph adenomas [74].

Zhang et al. explored the effect of exosomal lncRNA H19 on pituitary tumors. The same authors have noted in a previous study that the expression of lncRNA H19 is decreased in pituitary adenomas (PAs), and H19 overexpression could markedly inhibit the proliferation of pituitary tumor cells. They demonstrated that the expression level of exosomal H19 in all subtypes of pituitary tumors was significantly lower, and the exosomal H19 could be transported across the cell membrane to exert its inhibitory effect on pituitary tumor growth through inhibiting phosphorylation of the mTORC1 substrate 4E-BP1. Also, Cabergoline increased H19 expression and played a synergic therapeutic effect with exosomal H19. Therefore, exosomal H19 in prolactinoma patients may be a promising biomarker for predicting drug response [113].

The role and regulatory mechanism of tumor-derived exosomal AFAP1-AS1 (actin filament-associated protein 1 antisense RNA 1) in pituitary adenomas was the subject of a study by Tang et al. AFAP1-AS1 is a long non-coding RNA overexpressed in several cancers, playing a critical role in tumor proliferation. The data indicated that tumor-derived exosomes promote PA cell proliferation, migration, and invasion. Also, they modulate glucose metabolism by promoting glycolysis, facilitating the growth and invasion of PA cells. Exosomal AFAP1-AS1 plays an important role in promoting proliferation, migration, and invasion of the PA and facilitates energy metabolism in tumoral cells by its ability to modulate HuR protein stability. HuR is a protein that can act as an oncogene in various cancer types and is implicated in cell growth, angiogenesis, migration, invasion, glycolysis, and differentiation. This study demonstrates that AFAP1-AS1 may be a diagnostic marker in PA [137].

Exosome-derived circRNA was evaluated by Wan et al. The authors focused on the expression of circMFN2 in serum, exosomes, and tumor tissues of PitNETs and investigated this circRNA’s role in the tumors’ invasiveness. The study revealed a higher level of circMFN2 expression in invasive pituitary tumors, and this expression was significantly lower before and after surgery in the same patients. Also, there is evidence that circMFN2 could regulate miR-146a-3p to increase the proliferation and invasiveness of PA cells, partly through the TRAF6/NF-κB signaling pathway, a key player in regulating cell growth and gene transcription. Sustained activation of this pathway can lead to uncontrolled cell growth. Thus, circMFN2 can be a molecular marker for PA diagnosis and prognosis [138].

Chen et al. analyzed EMT-related markers in serum exosomes as potential diagnostic biomarkers for invasive PitNET. In the EMT process, immobile epithelial cells undergo a phenotypic transformation into mesenchymal cells that can invade and metastasize. There are changes in the expression of multiple markers: epithelial markers are downregulated, while mesenchymal markers (such as vimentin and N-cadherin) are upregulated. There is also a decrease in levels of strengthening intercellular adhesion proteins (E-cadherin and Epcam). The TGF-β/Smad signaling pathway is mainly involved in EMT. The authors decided to investigate Epcam, E-cadherin, N-cadherin, and key proteins of the TGF-β/Smads pathway (TGF-β, Smad3, Smad7) in invasive and non-invasive PitNET. In summary, they noted that the consistency of the EMT tendency in serum exosomes and tumor tissues suggests the potential of serum exosomes as an effective biomarker to evaluate the EMT tendency in pituitary adenoma and to diagnose invasive pituitary adenomas [139].

Ren et al. focused on diagnosing invasive non-functional pituitary adenomas using exosomal biomarkers. The authors evaluated the exosomes’ morphology and molecular composition isolated from invasive NF-PitNETs. They presented a novel concept of intercellular communication, according to which invasive NF-PitNETs cells specifically selected MMP1 (matrix metalloproteinase-1) mRNA and protein for uptake into the exosomal vesicle and transferred MMP1 activity to acceptor cells, including other tumor and vascular endothelial cells, that may well have vital functions in stimulating tumor invasion and angiogenesis. Increased expression of MMP1 and its formation in exosomes (exo-MMP1) were correlated with the invasiveness of NF-PitNETs [140].

These findings underscore exosomal molecular cargo potential as diagnostic and prognostic biomarkers. They can assist in early tumor detection, offer real-time insights into tumors’ evolution, and help refine and adjust treatment strategies.

A summary of the exosomal biomarkers in PitNETs and their clinicopathological relevance has been rendered in Table 6.

## 3. Liquid Biopsy in Patients with Suspected PitNETs or in the Postoperative Follow-Up

Throughout this comprehensive narrative review, information regarding the liquid biopsy in patients with suspected PitNETs or in the postoperative follow-up was provided. However, a special section extracting this information and focusing on these important aspects was considered relevant.

In patients with suspected PitNETs in which a diagnosis is needed, the first step is to assess if the tumor is functional or not, using hormonal panels. Consequently, a liquid biopsy can be taken into consideration if the neuroimaging aspects are equivocal or if the tissue biopsy is not feasible [18,23].

**Figure 3:**
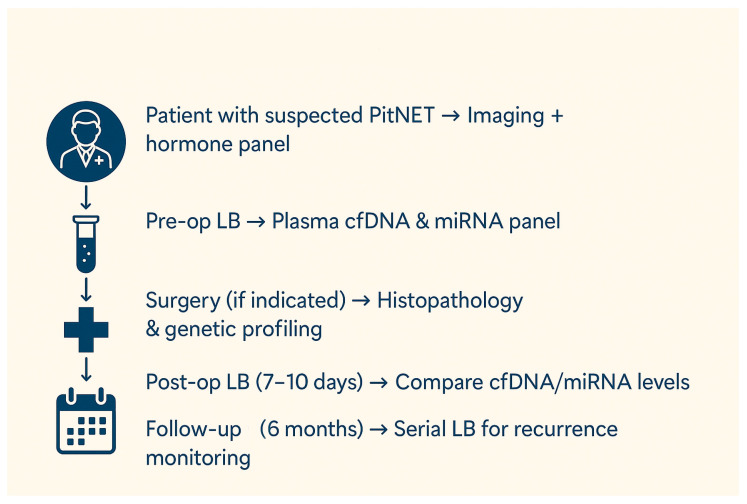
A schematic representation of the approach of liquid biopsy in patients with suspected PitNETs or in postoperative settings; LB—liquid biopsy.

In postsurgical follow-up, liquid biopsy can be used in addition to the neuroimaging examinations and hormonal testing to monitor residual disease or recurrences. In order to establish a preoperative baseline, a cfDNA/miRNA signature must be indicated. However, when it comes to early postoperative settings (7–10 days), persistent molecular signatures must be identified. When it comes to the periodic follow-up (e.g., every 6 months), the liquid biopsy should be considered, especially if residual tumor or high-risk histopathology is present [18,23,46]. Figure 3 summarizes the information provided about the approach of liquid biopsy in patients with suspected PitNETs or the postoperative follow-up.

## 4. Conclusions

Liquid biopsy has great potential in oncology as a minimally invasive approach, and it offers a valuable real-time perspective on distinct expression profiles in different types of tumors, including PitNETs. These data can be very useful for clinicians in screening, diagnosing, prognosticating, and monitoring disease progression and therapeutic response, as well as choosing a personalized treatment strategy. Many studies on genetic and molecular markers in PitNET tissue samples provide exciting information about tumor biology, but to date, specific studies on liquid biopsy biomarkers are still few. Over the past years, a certain understanding of the mechanisms involved in pituitary tumorigenesis has been gained, including the landscape of genomic alterations, changes in epigenetic profile, crucial molecules involved in specific signaling pathways, and non-coding RNA molecules with critical roles in malignant transformation. However, many studies regarding ncRNAs should be performed to complement the available data.

Although there are many issues in moving from basic to translational research, and it cannot replace tissue biopsy analysis, investigating the liquid biopsy biomarkers remains a promising tool in identifying distinctive genetic and molecular patterns of the PitNETs. Furthermore, it offers a non-invasive alternative to diagnostic and dynamic evaluation methods.

## Figures and Tables

**Table 1:** A summary of the characteristics, advantages, and disadvantages of the main components of liquid biopsy.

Biomarker	General Characteristics	Diagnostic Advantages	Diagnostic Limitations	Prognostic Advantages	Prognostic Limitations	Therapeutic Potential	Therapeutic Challenges
**CTCs**	Rare in PitNETs; detached tumor cells in blood; difficult to isolate due to EMT.	Potential early biomarker; detectable even in benign tumors.	Extremely rare; phenotypic changes impair detection.	May indicate metastatic potential if found.	Unclear utility; more studies needed.	Theoretically enables monitoring response to treatment.	No established protocols; research phase.
**ctDNA**	Short half-life; reflects tumor’s genetic/epigenetic profile; allows real-time monitoring.	Allows non-invasive genetic profiling; dynamic tracking.	Low concentration in benign tumors; technical challenges in detection.	Reflects tumor burden and heterogeneity; correlates with progression.	Limited clinical data; interpretation variability.	Changes post-treatment can indicate therapy efficacy.	Not validated in PitNET clinical practice.
**cfRNA**	Includes coding and non-coding RNAs (miRNA, lncRNA, circRNA); high stability in fluids.	Highly stable; specific expression profiles in subtypes.	Complex processing; not yet standard in clinical use.	Subtype-specific expression; potential to indicate aggression.	Role in prognosis still under investigation.	Can reveal resistance mechanisms (e.g., miR-93 in cabergoline resistance).	Many targets not yet clinically actionable.
**Exosomes**	Extracellular vesicles; carry nucleic acids, proteins; reflect tumor status and origin.	Easy to isolate; rich in specific biomarkers (e.g., miR, mRNA).	Requires advanced isolation methods; technical complexity.	Altered profiles (e.g., miR-486-5p) associated with recurrence risk.	Lack of standardization; needs validation.	Therapeutic targets (e.g., exosomal miR, mRNA) identified in resistant tumors.	Experimental stage; more validation required.

**Table 2:** Genetic alterations in PitNETs and their main characteristics.

Genetic Alteration/Syndrome	Clinicopathological Characteristics
*MEN1* mutation (MEN1 syndrome)	Associated with functional tumors; more invasive, aggressive, drug-resistant.
*CDKN1B* mutation (MEN4 syndrome)	MEN1-like phenotype; loss of cell cycle control, aggressive tumor behavior.
*PRKAR1A* mutation (Carney Complex)	Leads to GH-secreting tumors; linked to cAMP pathway activation and somatotroph tumorigenesis.
*SDHx/SDHAF2* mutations (3PAs syndrome)	Commonly PRL/GH-secreting or NF-PitNETs; aggressive with poor therapeutic response.
*DICER1* mutation (DICER1 syndrome)	Leads to rare tumors like pituitary blastoma in infancy; high aggressiveness.
*GPR101* duplication (X-LAG syndrome)	Early-onset GH/PRL-secreting tumors or hyperplasia; poor response to therapy.
*AIP* mutation (FIPA syndrome)	Aggressive GH-secreting tumors; treatment-resistant; early-onset.
*GNAS* mutation	Somatotroph tumors; increased cAMP and GH; smaller, less invasive, better SSA (somatostatin analogue) response.
*PTTG1* overexpression	Associated with increased proliferation and aggressiveness in various subtypes.
*STAT3* overexpression	GH hypersecretion; treatment resistance; aggressive behavior.
*CDH23* mutation	Somatotroph tumors; impaired adhesion; Wnt pathway involvement.
*SLC20A1* overexpression	Correlates with tumor size, recurrence, invasiveness in GH tumors.
*PRDM2* loss	Loss linked to GH tumorigenesis; affects c-Myc regulation.
*USP8* mutation	Corticotroph tumors; smaller size, higher ACTH; increased recurrence but better surgical outcome.
*USP48* mutation	Increases ACTH via NF-κB pathway; linked to corticotroph tumor progression.
*BRAF* mutation	Activates POMC transcription; corticotroph tumors; ACTH overproduction.
*TP53* mutation	Found in aggressive corticotroph tumors; poor prognosis.
*SF3B1* mutation	Prolactinomas; aberrant splicing affecting estrogen signaling; tumorigenic.
*HMGA1/HMGA2* overexpression	Overexpression in invasive GH and PRL tumors; chromatin regulation.
*PIK3CA* mutation	Promotes invasiveness; activates PI3K/Akt pathway in various PitNET subtypes.

**Table 3:** Genetic alterations by PitNET subtype and associated clinicopathological features.

Genetic Alteration/Syndrome	Associated PitNET Subtype	Clinicopathological Characteristics
*GNAS* mutation	Somatotroph	Increased cAMP and GH; smaller, less invasive; better response to SSAs.
*PTTG1* overexpression	Somatotroph	Linked to proliferation; aggressive tumors.
*STAT3* overexpression	Somatotroph	Leads to GH hypersecretion and therapy resistance.
*CDH23* mutation	Somatotroph	Impaired cell adhesion; Wnt pathway deregulation.
*SLC20A1* overexpression	Somatotroph	Correlated with tumor size, invasiveness, and recurrence.
*PRDM2* loss	Somatotroph	Loss affects c-Myc regulation; involved in tumorigenesis.
*USP8* mutation	Corticotroph	ACTH excess; better surgical remission but higher recurrence.
*USP48* mutation	Corticotroph	Promotes ACTH via NF-κB; progressive corticotroph tumors.
*BRAF* mutation	Corticotroph	Activates ACTH transcription; associated with corticotroph tumors.
*TP53* mutation	Corticotroph	Linked with poor outcomes; aggressive corticotroph tumors.
*SF3B1* mutation	Prolactinoma	Aberrant splicing; drives estrogen pathway in prolactinomas.
*MEN1* mutation	Various (familial)	Aggressive, drug-resistant tumors in familial cases.
*CDKN1B* mutation	Various (familial)	Loss of cell cycle control; MEN1-like phenotype.
*PRKAR1A* mutation	GH-secreting	Activates cAMP pathway; GH-producing tumors.
*SDHx/SDHAF2* mutations	PRL/GH-secreting or NF-PitNET	Aggressive, treatment-resistant; PRL or GH tumors.
*DICER1* mutation	Pituitary blastoma	Rare aggressive tumor in infants; pituitary blastoma.
*GPR101* duplication	GH/PRL-secreting or hyperplasia	Early-onset GH/PRL tumors; poor therapeutic response.
*AIP* mutation	GH-secreting	Early-onset, aggressive GH tumors; SSA resistance.
*PIK3CA* mutation	Multiple (ACTH, PRL, NF)	Increased invasiveness via PI3K/Akt; multiple subtypes.
*HMGA1/HMGA2* overexpression	GH and PRL PitNETs	Chromatin remodeling; overexpression in invasive GH and PRL tumors.

**Table 4:** Epigenetic alterations in PitNETs and their clinicopathological significance.

Epigenetic Alteration	Associated PitNET Subtype	Clinicopathological Characteristics
*NNAT* hypermethylation	Various subtypes	Loss of proliferation inhibition; found in ~70% of PitNETs.
*CDH13/CDH1* hypermethylation	Various subtypes	Loss of adhesion; associated with tumor aggressiveness.
*DAPK* gene silencing	Various (invasive tumors)	Linked to apoptosis evasion; more aggressive biological behavior.
*GADD45g* loss	Somatotroph/PRL-secreting	Loss of tumor suppressor gene; promotes growth in GH and PRL tumors.
*LGALS3* methylation	PRL-secreting	Oncogene activity; regulates migration, adhesion, and apoptosis.
*RASSF1A* hypermethylation	All subtypes (especially aggressive)	Correlated with high Ki-67 and aggressiveness.
*POMC* promoter demethylation	Corticotroph	Correlates with ACTH overproduction; USP8-mutant corticotrophs.
*FGFR2* methylation/*MAGE-3* hypomethylation	Corticotroph	*FGFR2* silenced (tumor suppressor); *MAGE-3* overexpressed (oncogene).
*TSP-1* downregulation (via miR-449c)	Corticotroph	TSP-1 suppresses proliferation; inhibited by miR-449c in Cushing’s disease.
*CDKN2A* promoter methylation	Gonadotroph, lactotroph, null cell PAs	Inactivation of p16 pathway; promotes proliferation and progression.
*MEG3* hypermethylation	Non-functioning	Loss of tumor suppressor; linked to progression and poor prognosis.
*ENC1* methylation	Non-functioning	Lower expression in invasive NFPAs; indicates aggressive behavior.
*FAM90A1* and *ING2* methylation	Non-functioning	Linked to recurrence risk in NF-PitNETs.

**Table 5:** Summary of cfRNAs involved in PitNETs with subtype associations and clinicopathological features.

cfRNAs	Associated PitNET Subtype	Clinicopathological Characteristics
miR-21 (exosomal)	Somatotroph	Promotes osteoblast proliferation in acromegaly; marker for disease activity.
miR-29c-3p	Somatotroph	Lower in uncontrolled acromegaly; potential monitoring marker.
miR-423-5p	Somatotroph	Reduces GH secretion; inhibits proliferation and migration in GH-secreting tumors.
miR-34a	Various	Tumor suppressor; downregulated in aggressive tumors.
miR-93	Prolactinoma	Mediates cabergoline resistance; regulates autophagy via ATG7.
miR-137	Prolactinoma/Non-functioning	Downregulates Wnt pathway; loss promotes invasiveness.
miR-9	Various	Promotes EMT; linked to aggressive phenotype.
miR-145-5p	Non-functioning/ACTH	Downregulation linked to invasiveness; potential TMZ sensitizer.
miR-122-5p	Corticotroph	Correlated with ACTH and treatment response in corticotroph tumors.
miR-486-5p (exosomal)	Non-functioning	Predicts recurrence in NF-PitNETs; MAPK pathway regulation.
miR-320a	Somatotroph	Downregulated in somatotrophs; marker of disease progression.
miR-143-3p	Gonadotroph	Reduced post-surgery; correlates with tumor behavior.
lncRNA MEG3	Non-functioning	Tumor suppressor; hypermethylated in NFPAs; loss linked to progression.
lncRNA HOTAIR	Non-functioning	Oncogenic role; upregulated in invasive NFPAs.
lncRNA H19	Prolactinoma/Somatotroph	Suppresses proliferation and enhances DA sensitivity; biomarker and target.
lncRNA RPSAP52	GH/PRL-secreting	Sponges miR-15a/16; promotes HMGA2; overexpressed in GH/PRL tumors.
lncRNA CLRN1-AS1	Prolactinoma	Suppresses Wnt pathway; acts as a tumor suppressor.
lncRNA THBS1	Corticotroph	Suppresses TSP-1; involved in Cushing’s disease progression.
lncRNA ANRIL	Invasive PitNET	Marker of invasiveness; elevated in invasive PitNETs.
lncRNA LINC00473	Invasive PitNET	Promotes proliferation via cyclin D1/CDK2; invasive tumors.
circOMA1	Non-functioning	Sponges miR-145-5p; promotes invasion in NFPAs.
circVPS13C	Non-functioning	Upregulated in high-risk NFPAs; downregulated post-op.
hsa_circ_0000066/hsa_circ_0069707	Non-functioning	Two-circRNA signature predicts recurrence.
hsa_circRNA_102597	Non-functioning	Downregulated in invasive tumors; potential severity marker.

**Table 6:** Summary of exosomal biomarkers in PitNETs and their clinicopathological relevance.

Exosome Biomarker/Component	Associated PitNET Subtype	Clinicopathological Significance
Exosomal mRNA: CDK6	Non-functioning	Upregulated in invasive NF-PitNETs; cell cycle regulator.
Exosomal mRNA: RHOU	Non-functioning	Upregulated in invasive NF-PitNETs; involved in cytoskeletal remodeling.
Exosomal mRNA: SPIRE2	Non-functioning	Linked to invasive behavior; actin nucleation function.
Exosomal miR-486-5p	Non-functioning	Most competent predictive biomarker for progression/recurrence; targets MAPK pathways.
Exosomal miR-423-5p	Somatotroph	Downregulated in somatotroph tumors; regulates GH and cell proliferation.
Exosomal miR-652-3p_R+1	Non-functioning	Altered in NF-PitNETs; potential diagnostic marker.
Exosomal miR-1180-3p	Non-functioning	Altered in NF-PitNETs; prognostic significance unclear.
Exosomal miR-151a-5p	Non-functioning	Altered in NF-PitNETs; contributes to the exosomal signature.
